# Development and characterization of a combined fluorescence and spatial frequency domain imaging system for real-time dosimetry of photodynamic therapy

**DOI:** 10.1117/1.JBO.30.S3.S34103

**Published:** 2025-05-28

**Authors:** Alec B. Walter, E. Duco Jansen

**Affiliations:** a Vanderbilt University, Department of Biomedical Engineering, Nashville, Tennessee, United States; b Vanderbilt University, Biophotonics Center, Nashville, Tennessee, United States; c Vanderbilt University Medical Center, Department of Neurosurgery, Nashville, Tennessee, United States

**Keywords:** spatial frequency domain imaging, photodynamic therapy, dosimetry, fluorescence, porphyrin

## Abstract

**Significance:**

Current methods of measuring dosimetry for photodynamic therapy (PDT) have proven to be inadequate in their inability to provide accurate, real-time, and spatially resolved monitoring without interrupting the PDT treatment.

**Aim:**

Our goal was to develop and validate a combined treatment and dosimetry system capable of monitoring implicit and explicit dosimetry in real time during non-contact PDT.

**Approach:**

By employing both fluorescence imaging and spatial frequency domain imaging (SFDI), designed with low-cost, off-the-shelf components, the combined imaging system would be able to provide information on the spatial distributions of photosensitizer concentrations, tissue oxygenation, and delivered light dose, all while monitoring the photobleaching dynamics of the photosensitizer. Although the concept behind the combined system is not specific to any one photosensitizer, we focused on designing the system for the endogenous PDT of Gram-positive bacteria which utilizes coproporphyrin III as the photosensitizer.

**Results:**

The overall performance of the system was assessed, with the accuracy, precision, and resolution of the SFDI-derived optical property maps being determined to fall within comparable ranges to other systems, despite the $1.0\;{\mathrm{mm}}^{-1}$ spatial frequency utilized for the shorter wavelengths. After validating the ability of the system to correct for tissue-like optical properties, and thus produce accurate quantitative fluorescence images, a preliminary assessment of antimicrobial PDT photobleaching dosimetry was performed, and high correlations were found between the fluorescence and PDT outcomes.

**Conclusions:**

Overall, the developed imaging system showcases the potential to enable a more thorough analysis of PDT dosimetry and the impact of different variables on treatment outcomes.

## 1 Introduction

One of the major limiting factors in developing effective clinical treatments utilizing photodynamic therapy (PDT) is the lack of accurate and predictive dosimetry. In a broad sense, dosimetry involves the planning and verification process to determine that the appropriate level of treatment has been administered to the tissue of interest; too low of a dose and the targeted cells will survive, but too high and the surrounding healthy tissue can be negatively affected or damaged.^1^^,^^2^ The active agent in PDT is reactive oxygen species (ROS), which is generated at the micro-environment level though a complex interaction of photosensitizer (PS) concentration, tissue oxygenation, and light distribution. As these three are both interdependent and dynamic, the cumulative treatment dose is often highly variable both spatially within the tissue and across subjects.^3^^,^^4^ This is an issue as most PDT clinical trials assess dosimetry by specifying up to three fixed inputs: the photosensitizer dose per kilogram body weight, the light dose at the surface of the tissue, and the amount of time between PS administration and light application.^5^ Although these parameters allow for consistent administration of treatment, it does not address any of the inherent variability that may occur, thus leading many of these trials to report highly variable response rates with the treatment efficacy varying greatly among patients. With no manner of determining why one patient responded to treatment while another did not, clinicians have been hesitant to widely adopt PDT.^1^ Although there are multiple dosimetry metrics that have been shown to strongly correlate with treatment outcome, the use of these metrics in practical cases has been limited due to the difficulties in measuring the necessary parameters while being minimally invasive, undisruptive to treatment, and easy to use. Thus, there is a need to provide intuitive and non-interruptive dosimetry measures to clinicians that can allow them to monitor and predict the response to PDT therapy.

Due to the difficulty of directly measuring the production of ROS within tissue, most PDT dosimetry takes the form of one of two different types of indirect methods, implicit and explicit dosimetry.^1^^,^^5^^,^^6^ The goal behind implicit dosimetry is to utilize a single, easily measurable metric that integrates together all of the individual parameters that influence the outcome of photodynamic therapy, thus accounting for most, if not all, of the inherent variability. The most common form of implicit dosimetry is accomplished through monitoring the photobleaching of the PS.^1^^,^^5^ Many PSs are fluorescent, and over the course of PDT treatment, they undergo irreversible photobleaching which results in a decrease in the fluorescence intensity. As photobleaching can occur through ROS-dependent mechanisms, the loss in PS fluorescence can be due to their own generation of singlet oxygen and is thus dependent on the same complex interactions of light, PS, and oxygen that determine the effective PDT dose.^7^^–^^16^ However, the two useful photobleaching metrics, the rate and the cumulative amount, are both dependent on the physiological state of the tissue, with the rate of photobleaching being dependent on the state of tissue oxygenation, whereas the cumulative amount of photobleaching has only been found to be predictive when taken as the absolute quantity of PS that has photobleached, necessitating the need to have *a priori* knowledge of the localized PS concentrations.^7^^–^^9^^,^^12^^–^^14^ Unlike implicit dosimetry, explicit dosimetry attempts to avoid this dependency by individually measuring one or more of the three photodynamic inputs of light, photosensitizer, and oxygen before using them to estimate the amount of singlet oxygen produced. This can be accomplished either through a direct correlation or by integrating them together into a more complex model of ROS dynamics.^5^^,^^17^^–^^19^

Although both implicit and explicit dosimetry have been shown to be effective strategies in predicting treatment outcomes, the use of these metrics in practical cases has been hampered due to the different measurement techniques requiring interruption of the treatment to use a secondary measurement device while also not accounting for any spatial heterogeneities that may be present within the tissue of interest. In addition, almost all practical dosimetry metrics require additional information about the tissue, or another dosimetry parameter, to be accurate. Thus, for a PDT dosimetry system to be clinically viable, it will need to be both integrated into the therapeutic system, to allow for minimal interruptions to the treatment, and provide multiple types of spatially resolved dosimetry measures.

We hypothesize that a combination of wide-field fluorescence imaging and spatial frequency domain imaging (SFDI) would allow for the monitoring of a number of different spatially resolved implicit and explicit dosimetry parameters throughout non-contact PDT treatment. SFDI is a wide-field imaging modality that uses diffuse reflectance images to produce spatial maps of the absorption and reduced scattering coefficients.^20^^,^^21^ This is accomplished by projecting a two-dimensional sinusoidal pattern of light onto the sample and analyzing how the intensity of the reflected pattern changes as a function of spatial frequency. By measuring the reflectance using at least two spatial frequencies, the optical properties can be rapidly determined using an inverse model. By performing this process in a pixel-wise manner, spatially resolved maps of the absorption and reduced scattering coefficients can be obtained.

With this combination, fluorescence imaging provides information on the photosensitizer concentration and photobleaching rate, whereas the optical properties maps obtained through SFDI allow for the calculation of the sample-dependent light fluence, tissue oxygenation, and the correction values used to compensate for the effect that the tissue has on the measured fluorescence, allowing for a quantitative assessment of PS concentration. Although fluorescence imaging can be performed concurrently with PDT, as the treatment light acts as the excitation light, treatment will need to be temporarily halted to acquire any SFDI measurements. In traditional SFDI, the captured images are demodulated through a phase-stepping approach that requires a minimum of six images to be taken for each wavelength of interest, a process that would lead to unacceptably long interruptions during PDT.^20^ To ensure that any interruption to the therapeutic light dose is negligible, a single-frame variant of SFDI, which uses Fourier-domain separation of zero (DC) and non-zero (AC) spatial frequency components, called single snapshot of optical properties (SSOP), will be utilized.^22^ SSOP has proven capable of providing video rate assessment of tissue optical properties and has been shown to match the acquisition speed of fluorescence imaging.^23^^–^^27^ This would allow for the periodic replacement of a few fluorescence frames with a set of SFDI reflectance images, minimizing any impact on the ongoing treatment.

The goal of this study was to design, develop, and validate a combined imaging system that uses low-cost, commercial off-the-shelf components and is capable of leveraging photosensitizer fluorescence and SSOP to provide spatially and temporally resolved dosimetry while minimally interrupting treatment. This involved characterizing the accuracy, precision, and resolution of the SFDI portion of the system before assessing the ability of the combined system to yield the relative dosimetry parameters in real time. Although the concept behind such a combined system is not specific to any one photosensitizer, this work focused on designing the system for the endogenous antimicrobial PDT (aPDT) of Gram-positive bacteria, building off of our previous work.^28^^–^^30^ As such, the ability to produce quantitative fluorescence images of coproporphyrin III (CPIII), the endogenous photosensitizer produced by Gram-positive bacteria, was determined before performing an initial investigation into the dosimetry relationships present during aPDT treatment of methicillin-resistant *Staphylococcus aureus.*

## 2 Materials and Methods

### 2.1 SFDI System Design

**Fig. 1:**
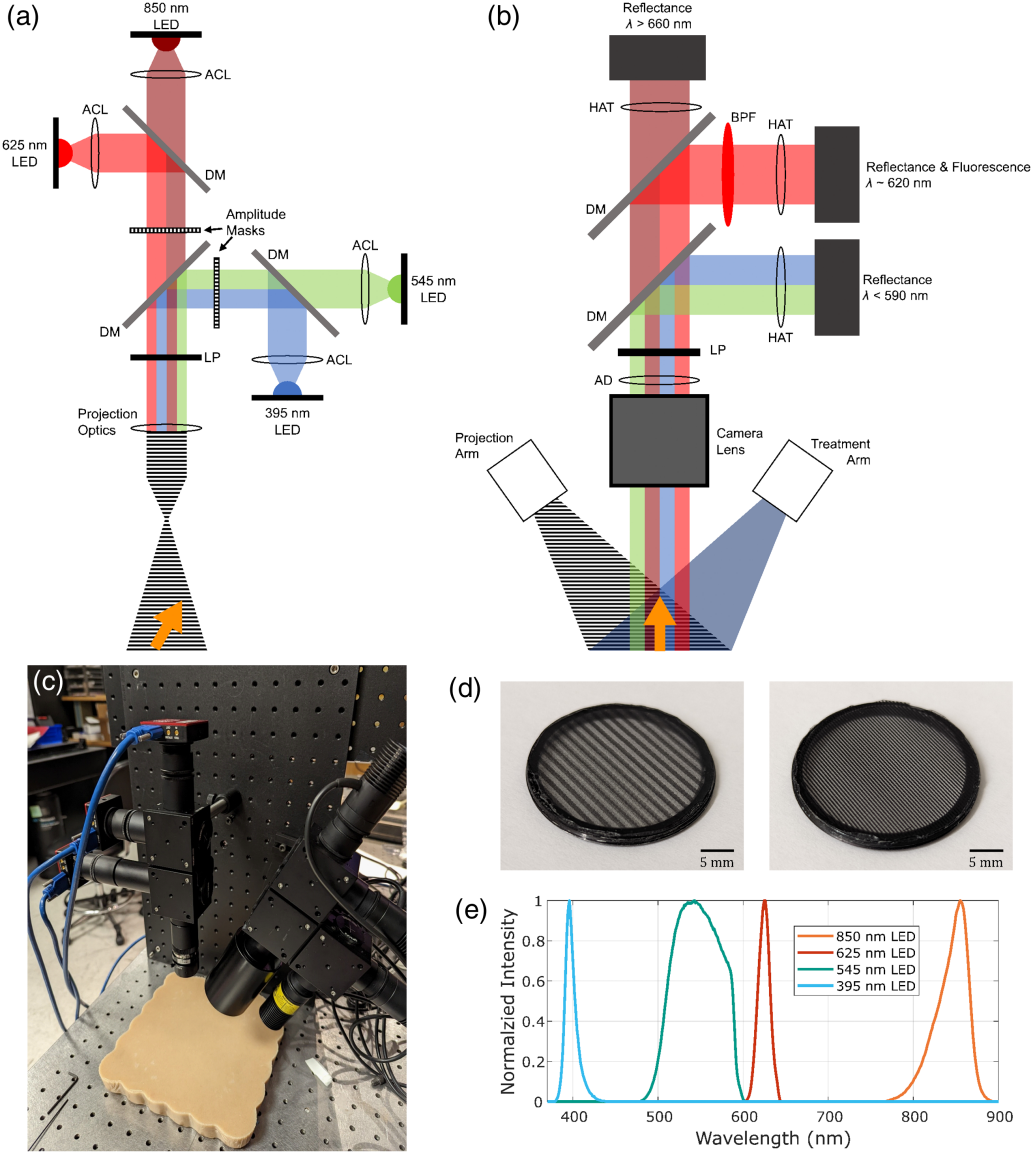
Schematic of the combined SFDI and fluorescence imaging system for aPDT dosimetry. (a) Projection arm of the system provides spatially modulated light at four different wavelengths (395, 545, 625, and 850 nm) using two independent amplitude masks. The light is spatially combined and passed through a removable linear polarizer before being projected onto the sample, indicated with an orange arrow, at an angle. (b) Separately, the treatment arm delivers the light to the sample for PDT treatment and fluorescence excitation. The resulting reflectance and fluorescence signals are collected by the imaging arm at three different channels, λ<590  nm, 590  nm≤λ≤660  nm, and λ>660, where the middle channel is further cleaned up with a bandpass filter. (c) Picture of the combined imaging system. (d) Pictures of the printed amplitude masks used for $0.3\;{\mathrm{mm}}^{-1}$ (left) and $1.0\;{\mathrm{mm}}^{-1}$ (right) patterned illumination. (e) Normalized intensity profiles of the four projection LEDs at their respective detectors. ACL, aspheric condenser lens; DM, dichroic mirror; LP, linear polarizer; AD, achromatic doublet; BPF, bandpass filter; HAT, Hastings achromatic triplet.

In this work, a custom imaging system capable of both spatial frequency domain imaging and fluorescence imaging was designed and validated. The general setup of the system is presented in Fig. 1. The goal of the combined system is to be able to perform three separate tasks during endogenous aPDT: supply the therapeutic light to the tissue, collect the fluorescence emitted by the porphyrin photosensitizer, and provide spatially resolved optical property maps at several different wavelengths for dosimetry assessment. This is accomplished using two separate illumination arms, one providing illumination for the PDT treatment and fluorescence excitation and the other providing the patterned illumination required for SFDI. The treatment arm consists simply of a high-power light-emitting diode (LED) which is collimated with a 20 mm aspheric condenser lens (ACL2520U-A, Thorlabs, Newton, New Jersey, United States) and cleaned up with a bandpass filter corresponding to the wavelength of the LED. This LED and filter pair can be swapped easily to target different photosensitizer excitation peaks, with a 395 nm LED (M395L5, Thorlabs) and a 400 nm bandpass filter (FBH400-40, Thorlabs) being used for this work to target the primary absorption peak of CPIII.^29^

The patterned illumination arm, as seen in Fig. 1(a), consists of four different channels each consisting of a high-powered LED light source thermally bonded to a heatsink to minimize any thermal drift in the output power and peak wavelengths. Two of these channels correspond to possible wavelengths used in the treatment arm, 395 and 545 nm (M565L3, Thorlabs), thus providing the optical properties at the utilized excitation wavelength. These are used further in a portion of the calculation to determine the quantified, media-corrected fluorescence of the photosensitizer, described below. The third channel uses an LED which corresponds to the peak emission wavelength of the photosensitizer, which for CPIII is at 620 nm (M617L3, Thorlabs). Measurements at this wavelength complete the quantitative fluorescence calculation, while also being used alongside the fourth channel, at 850 nm (M850L3, Thorlabs) to measure the ratio of oxy- and deoxyhemoglobin and thus estimate the oxygenation state of tissue samples.^31^^,^^32^ Each of these LEDs is powered using drivers with built-in triggering capabilities to allow for automated control of the illumination. The light from each of the LEDs is collected and collimated using a 20 mm focal length, aspheric condenser lens before being spatially combined with three long-pass dichroic mirrors with edge wavelengths of 470 nm (FF470-Di01, Semrock, West Henrietta, New York, United States), 605 nm (FF605-Di02, Semrock), and 660 nm (FF660-Di02, Semrock), respectively.

The sinusoidal illumination required for SFDI is accomplished by placing amplitude masks in the back focal planes of the projection optics, resulting in each pair of LEDs sharing a mask. As seen in Fig. 1(d), the amplitude masks were made by printing binary images onto transparency film using a commercial laser jet printer and bonding the resulting masks onto SM1 retaining rings for easier placement and alignment within the system. Pseudo-sinusoidal patterns were created from the binary printing through a pixel dithering process, which served to dampen any harmonics that would persist in the collected reflectance. The high-frequency pattern that arose from the dithering process was found to be sufficiently attenuated by the low-pass nature of scattering samples and thus was not found to impact the collected reflectance.

Prior to being projected onto the sample, the modulated and spatially combined light is passed through a linear polarizer (LPNIRE100-B, Thorlabs) with an orthogonal orientation with respect to the matching polarizer in the imaging arm of the system. Although the cross-polarization, along with the 40-deg angle of incidence of the illumination arm, serves to reduce the impact of specular reflectance on the collected reflectance images, it was specifically chosen to suppress the effects of sub-diffuse backscattering, as showcased in our previous work.^33^ This would allow for any samples with uncategorized scattering behavior to be accurately measured using a single-term Henyey–Greenstein approximation of the scattering phase function. To guarantee sufficient cross-polarization at all four wavelengths, the extinction ratio of the polarizers was assessed through a rotation test performed with the auto-polarizer of the universal measurement accessory (Agilent, Santa Clara, California, United States) of the Cary 5000 spectrophotometer (Cary 5000, Agilent) and was found to be maintained above 200:1 for all four wavelengths of interest. Although these polarizers are reported to have a degree of internal scattering and are not recommended for imaging applications, we did not observe any decrease in image quality due to their inclusion.

The imaging arm of the system is situated orthogonally with respect to the sample, with the general layout shown in Fig. 1(b). Light is collected with a 12 mm fixed focal length camera lens (MVL12WA, Thorlabs) before being collimated with a 50 mm focal length achromatic doublet (AC254-050-AB, Thorlabs). This collimated light is passed through the previously mentioned linear polarizer before being spectrally separated into three spatially aligned channels with long-pass dichroic mirrors. Using cutoff wavelengths at 596 nm (FF596–Di01, Semrock) and 660 nm (FF660-Di02, Semrock), the peak fluorescence emission of the porphyrin photosensitizer, which for CPIII is located near 620 nm, and the corresponding reflectance signal are separated from the shorter visible wavelengths, which include the reflectance of the excitation wavelengths, and the longer NIR wavelengths, which capture the 850 nm reflectance. To ensure a clean fluorescence signal, an additional bandpass filter was inserted in the fluorescence channel (FF01–618/50, Semrock). The collimated light of each channel is re-imaged onto a 1440×1080 monochrome CMOS camera (CS165MU1, Thorlabs) using a 40 mm focal length Hastings achromatic triplet (TRH254-040, Thorlabs). This results in a total imaging area of 62  mm×46.5  mm, with each pixel representing 43  μm×43  μm square in the sample plane. The spectral throughput of the system was assessed by measuring the spectral profiles of each LED at their corresponding detectors [Fig. 1(e)] using a miniature spectrometer (Flame-S-VIS-NIR, Ocean Insight, Orlando, Florida, United States), with the light from the illumination arm being reflected off of a 99% diffuse reflectance standard (USRS-99-020, LabSphere, North Sutton, New Hampshire, United States) to allow for collection by the imaging arm without further influencing the spectral response.

### 2.2 Spatial Frequency Determination

To determine the spatial frequencies for each wavelength pair that would result in the most robust determination of tissue optical properties, direct simulations of SFDI were performed on a model of the human skin using the MCXLAB version of the Monte Carlo eXtreme.^34^ The simulated skin model is similar to those reported for predicted PDT efficacy in depth, consisting of seven separate layers representing the stratum corneum, living epidermis, papillary dermis, subpapillary plexus, reticular dermis, cutaneous plexus, and subcutaneous adipose tissue.^29^ For each layer, the thickness was selected based on average reports for healthy adults (Table 1).^35^ In addition, the optical properties for each wavelength of interest were determined using the work of Meglinski and Matcher.^36^ This layer-based differentiation was used to provide a better prediction of the expected reflectance as varying optical properties and spatial frequencies alter the effective penetration depth of the light.^20^

**Table 1:** Layer properties for the Monte Carlo model of skin.

		395 nm		545 nm		625 nm		850 nm		g	n
	d (mm)	$\mu_{a}$ (${\mathrm{mm}}^{-1}$)	$\mu_{s}^{\prime }$ (${\mathrm{mm}}^{-1}$)	$\mu_{a}$ (${\mathrm{mm}}^{-1}$)	$\mu_{s}^{\prime }$ (${\mathrm{mm}}^{-1}$)	$\mu_{a}$ (${\mathrm{mm}}^{-1}$)	$\mu_{s}^{\prime }$ (${\mathrm{mm}}^{-1}$)	$\mu_{a}$ (${\mathrm{mm}}^{-1}$)	$\mu_{s}^{\prime }$ (${\mathrm{mm}}^{-1}$)		
Stratum corneum	0.02	1.370	8.31	0.547	3.24	0.375	2.28	0.185	1.24	0.86	1.50
Living epidermis	0.08	1.944	8.31	0.699	3.24	0.444	2.28	0.169	1.24	0.80	1.34
Papillary dermis	0.15	1.190	4.62	0.274	3.18	0.100	2.70	0.015	1.88	0.80	1.40
Subpapillary plexus	0.08	3.096	0.77	0.564	0.53	0.107	0.45	0.028	0.31	0.95	1.39
Reticular dermis	1.50	0.936	4.62	0.191	3.18	0.053	2.70	0.014	1.88	0.80	1.40
Cutaneous plexus	0.08	3.252	0.77	0.562	0.53	0.080	0.45	0.021	0.31	0.95	1.38
Subcutaneous tissue	1.09	1.080	1.54	0.214	1.06	0.054	0.90	0.014	0.63	0.75	1.44

**Fig. 2:**
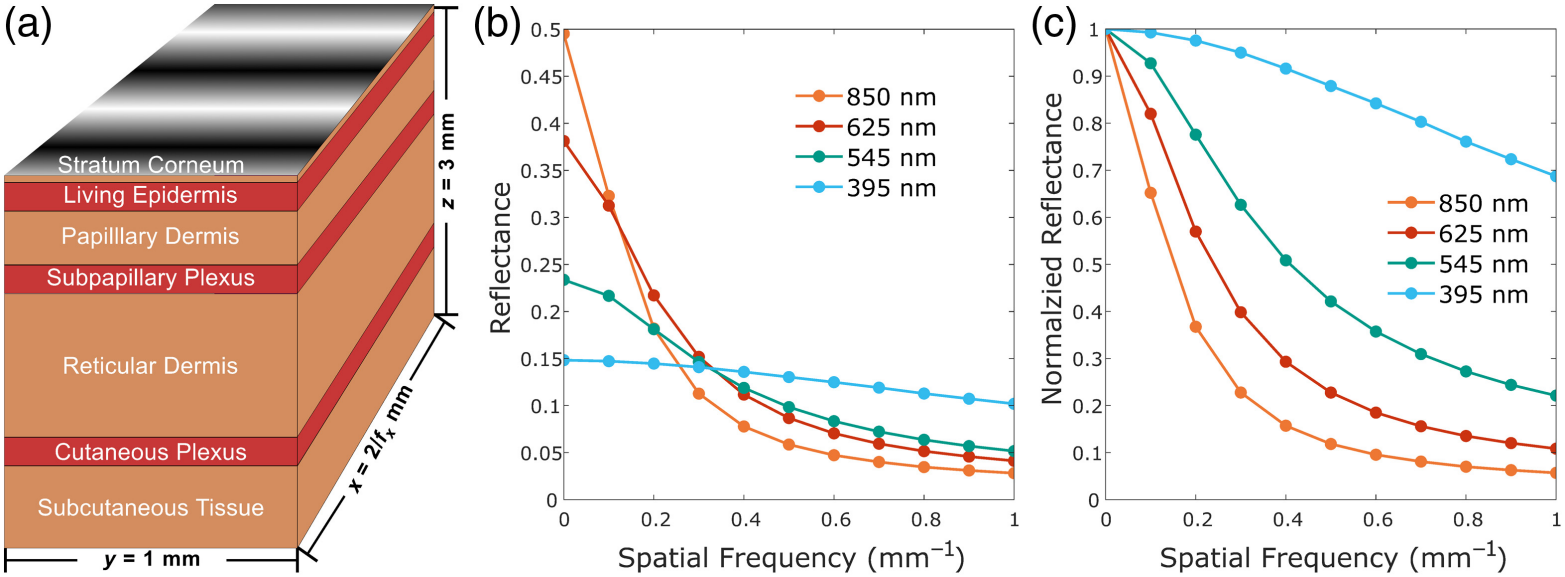
Predicted spatial frequency–dependent reflectance of the human skin. SFDI Monte Carlo simulations were performed using a seven-layer human skin model (a) with a fixed width (1 mm) and depth (3 mm) and a variable length such that two full periods of the patterned illumination were always applied. The thickness (d, mm), scattering anisotropy (g), and refractive index (n) for each layer were fixed, whereas the absorption ($\mu_{a}$, ${\mathrm{mm}}^{-1}$) and reduced scattering ($\mu_{s}^{\prime }$, ${\mathrm{mm}}^{-1}$) coefficients were varied for the four illumination wavelengths. The absolute (b) and normalized (c) Monte Carlo results for the reflectance of the seven-layer model with spatial frequencies between 0 and $1.0\;{\mathrm{mm}}^{-1}$ at the four illumination wavelengths.

For the simulation of SFDI, the modeled volume of skin [Fig. 2(a)] was illuminated by a $10^{8}\;\text{photon}$, sinusoidally patterned source using one of 10 equally spaced spatial frequencies from 0.1 to $1.0\;{\mathrm{mm}}^{-1}$. To resolve the thinner layers of the model, a fixed voxel size of 20  μm was used. To reduce memory constraints and increase the overall speed of the simulation, the length of the volume was set to scale with the applied spatial frequency so that two full periods would always be applied, whereas the width and depth were fixed at 1 and 3 mm, respectively. Although this depth did not allow for the full thickness of the subcutaneous skin layer, it was found to be sufficiently optically thick for all of the performed simulations. It has been previously shown that for 850 nm light, which is the least attenuated wavelength used in the combined system, the fluence rate in the skin can be expected to reach 10% of its starting value at around 3.5 mm in depth.^37^ As any detected photons would have to pass back through that thickness of skin, photons originating from below the 3 mm simulation thickness are unlikely to contribute significantly to the overall DC reflectance, let alone the AC reflectance. To approximate the limited size of the volume and source as semi-infinite, the boundary conditions on all four x and y faces were set to be cyclical, with photons exiting the volume from one face re-entering from the opposite face. Although the optical properties of each layer were defined spectrally with a 1 nm step size, the properties at a single wavelength do not serve to accurately represent the behavior of the non-monochromatic LED light sources. As such, the properties expected to be seen by each of the imaging channels in the SFDI system were determined by taking the weighted average of the spectral properties using the normalized spectral profiles of each illumination LED as the weighting functions (Table 1).

For each modeled spatial frequency, three different phase shifts, of 0, 2π/3, and 4π/3, were used, and the spatially resolved diffuse reflectance was recorded for each. After normalizing to the average intensity of the illumination pattern, the AC reflectance for each spatial frequency was determined using the standard three-phase demodulation approach, whereas the DC reflectance was approximated by averaging all three phases together and averaging those results across all simulated spatial frequencies, as seen in Fig. 2(b).^20^ From these results, it can be seen that the reflectance behaviors for the 850, 625, and 545 nm LEDs were similar, with a relatively sharp drop off in reflectance with increasing spatial frequency. With this type of behavior, an ideal spatial frequency to use for single-phase SFDI would be around $0.3\;{\mathrm{mm}}^{-1}$. This is because the frequency is large enough to sufficiently separate it from the DC signal during Fourier domain demodulation while maintaining an AC reflectance signal at least 20% that of the matching DC reflectance, as seen in Fig. 2(c).^23^ However, although a $0.3\text{-}{\mathrm{mm}}^{-1}$ spatial frequency works cleanly for the 850 and 625 nm channels, the 545 nm channel shares an amplitude mask with the 395 nm channel which displayed a drastically different reflectance response. Due to the much higher scattering and absorption, a significant decrease in the AC reflectance was only observed at very high spatial frequencies. When the DC and AC reflectance values are too close to one another, the inverse models used to obtain the absorption and reduced scattering values enter an exponential region where slight variances in the reflectance values from noise can result in extreme changes in optical properties, ultimately leading to inaccuracies. In addition, when too close together, measurement noise can result in pixels with measured AC reflectance greater than the paired DC reflectance, which cannot exist due to the low-pass behavior of scattering media. Thus, a spatial frequency of $1.0\;{\mathrm{mm}}^{-1}$ was chosen as it was predicted to provide at least a 30% difference in the DC and AC reflectance at 395 nm while maintaining an AC reflectance above 20% of the DC reflectance for the 545 nm channel.

### 2.3 SFDI Acquisition and Processing

Reflectance images were acquired using a variable exposure time such that the greatest pixel intensity fell within 80% of the maximum value of the detector. To bring the resulting images back onto the same relative scale, pixel intensities were normalized to their respective exposure times. To minimize the effects of ambient light on the measurements, background images with all LEDs off were taken using the same exposure time and were subtracted from the sample images before use. Image processing, described below, was performed in MATLAB on the background-subtracted images after conversion into double-precision arrays.

Following the generalized procedure for the SSOP approach to SFDI, single frames of patterned reflectance were demodulated into their corresponding DC and AC components using Fourier domain filtering. For the $0.3\text{-}{\mathrm{mm}}^{-1}$ images, demodulation was accomplished using the set of two-dimensional (2D) anisotropic filters described and recommended by Aguénounon et al.^23^ This included a sine bandpass filter to isolate the DC component and a Blackman high-pass filter to separate the AC component. To suppress any potential harmonic frequencies that would be present due to the imperfect nature of the amplitude masks, notch filters made from inverted sine bandpass filters were included at the second and third harmonics of the detected AC frequency. For the $1.0\text{-}{\mathrm{mm}}^{-1}$ images, generalized spectral notch filters were used instead.^38^^–^^40^ In these filters, the location and intensity of the peak frequencies along the x-direction are found, and unwanted frequencies are removed by thresholding all pixels within a specified distance and intensity range of the peaks. For the DC demodulation filter, the positive and negative AC frequencies were removed, along with the first pair of harmonics if still detectable. For the AC demodulation filter, the DC peak, the negative AC frequency, and both the first and second pair of harmonics, if detected, were removed. To reduce any filtering artifacts, the spectral notch filters were smoothed with a 2D Gaussian filter, with a standard deviation of 1, prior to use.

Independent of the filters used, the demodulation process yields two images, $M(p_{xy},f_{x})$, where the value at each pixel, $p_{xy}$, corresponds to the DC ($f_{x}=0\;{\mathrm{mm}}^{-1}$) and AC ($f_{x}>0\;{\mathrm{mm}}^{-1}$) intensities of the sample. To convert these intensity images into the corresponding reflectance images, the effects of the system modulation transfer function must be corrected. This was accomplished by normalizing the sample images to a reference phantom of known optical properties, such that

$$R_{d}(p_{xy},f_{x})=\frac{M(p_{xy},f_{x})}{M_{\mathrm{ref}}(p_{xy},f_{x})}R_{d,\mathrm{ref},\mathrm{pred}}(f_{x}),$$ (1)

where $M_{\mathrm{ref}}$ is the demodulated intensity image of the reference, and $R_{d,\mathrm{ref},\mathrm{pred}}$ is the reflectance of the reference, at the corresponding spatial frequency, predicted from its optical properties. The reference phantom used with the system was a circular phantom with a diameter larger than the illumination spot size, created using our previously developed multi-pigment phantom approach.^41^ The absorption coefficients of the reference were $\mu_{a}=0.077$, 0.172, 1.105, and $2.702\;{\mathrm{mm}}^{-1}$, whereas the reduced scattering coefficients were $\mu_{s}^{\prime }=1.566$, 2.199, 2.710, and $3.875\;{\mathrm{mm}}^{-1}$ for the imaging wavelengths of 850, 625, 545, and 395 nm, respectively. In this work, the corresponding reference reflectance values were obtained using a previously developed, custom, logarithmically spaced, white Monte Carlo model.^33^

Optical property maps of the absorption and reduced scattering coefficients are obtained from the pair of reflectance images using a set of look-up tables (LUTs). The LUTs are computed from the white Monte Carlo model and map what optical properties correspond to a given pair of reflectance values at the specified spatial frequencies. The utilized LUTs are logarithmically sampled with respect to the reflectance values, which improves the accuracy at lower reflectance pairs. For an accurate inverse solution, the utilized LUTs were computed using a white Monte Carlo model with a refractive index matched to an estimate of the sample and a scattering phase function matched to both the sample and the polarization state of the imaging system.^42^ With the SFDI portion of the imaging system being cross-polarized, the white Monte Carlo model and the optical property LUTs used a forward-scattering, single-term Henyey–Greenstein scattering phase function with an overall anisotropy of $g_{1}=0.985$.^33^ As the LUTs are sparsely sampled, the optical properties for an arbitrary pair of measured DC and AC reflectance values are determined through a bilinear interpolation of the LUT pair. Repeating this process in a pixel-wise manner yields the spatial maps of optical properties.

### 2.4 SFDI System Characterization

To characterize the performance of the SFDI system, the accuracy, precision, and resolution of the produced optical property maps were determined. Accuracy, in this context, refers to the ability of the SFDI system to correctly determine the optical properties of unknown samples and can be impacted by a number of different factors including the type of demodulation used and the accuracy of the utilized reflectance model.^33^^,^^42^ To determine the accuracy of the constructed system, a series of 16 epoxy resin phantoms were made, and their ground truth optical properties were determined using integrating sphere measurements and an inverse Monte Carlo approach (see Table 2). As described in more detail in our previous works, these homogenous phantoms were made using a 35×35  mm mold, had an average thickness of 17 mm, and were made using four different scattering levels and four different absorption levels, with a range of the absorption coefficients varying at each wavelength channel to match the range expected to be found in tissue.^33^^,^^41^ Each of the 16 phantoms was imaged using all four wavelengths, and from the resulting optical property maps, a 13×13  mm (300×300  pixels) region of interest in the center of the phantom was assessed by determining the average value, as well as the upper and lower quartiles of the distribution, and comparing it to the previously determined ground truth optical properties. The accuracy of the measurements was determined through the mean absolute percentage error (MAPE) of the region of interest, averaged across the phantoms for each wavelength.

**Table 2:** Ground truth optical properties of the multi-pigment epoxy phantoms.

Phantom	850 nm		620 nm		545 nm		395 nm	
	$\mu_{a}$ (${\mathrm{mm}}^{-1}$)	$\mu_{s}^{\prime }$ (${\mathrm{mm}}^{-1}$)	$\mu_{a}$ (${\mathrm{mm}}^{-1}$)	$\mu_{s}^{\prime }$ (${\mathrm{mm}}^{-1}$)	$\mu_{a}$ (${\mathrm{mm}}^{-1}$)	$\mu_{s}^{\prime }$ (${\mathrm{mm}}^{-1}$)	$\mu_{a}$ (${\mathrm{mm}}^{-1}$)	$\mu_{s}^{\prime }$ (${\mathrm{mm}}^{-1}$)
1	0.018	0.352	0.031	0.536	0.159	0.762	0.765	1.331
2	0.013	1.040	0.026	1.480	0.138	1.614	0.434	2.626
3	0.013	1.593	0.035	2.140	0.179	2.588	0.383	3.969
4	0.019	2.021	0.034	2.895	0.111	3.518	0.287	5.403
5	0.048	0.385	0.116	0.534	0.374	0.752	1.204	0.886
6	0.046	0.996	0.086	1.393	0.244	1.579	0.820	2.967
7	0.048	1.599	0.094	2.270	0.337	2.709	0.871	4.074
8	0.059	2.283	0.094	3.009	0.281	3.641	0.682	5.523
9	0.099	0.361	0.180	0.530	1.086	0.767	2.656	1.592
10	0.083	0.905	0.204	1.406	1.107	1.518	2.373	2.533
11	0.077	1.566	0.172	2.199	1.105	2.710	2.972	4.069
12	0.087	2.145	0.167	2.963	1.035	3.776	2.571	5.794
13	0.119	0.295	0.309	0.559	2.452	0.717	3.859	1.132
14	0.097	0.903	0.232	1.395	2.566	1.597	4.206	2.380
15	0.141	1.469	0.294	2.109	2.492	2.504	5.308	3.854
16	0.142	2.158	0.240	3.115	2.696	3.554	5.093	5.136

The precision of an SFDI system typically refers to the ability of the system to produce consistent measurements over time and is assessed through a drift test.^43^^,^^44^ A drift test involves taking repeated measurements on the same phantom and determining the general variability over time, as well as any potential time-dependent shifts that occur in the measured optical properties. For this system, the precision of each channel was determined using a 90-min drift test, with measurements taken every 5 min. Similar to the accuracy assessment, a 13×13  mm region of interest was analyzed with the average optical properties within the region of interest being determined. These values are compared with the average over 90 min, with both the standard deviation and coefficient of variation, or relative standard deviation, for each wavelength being determined. This process was repeated for a subset of six phantoms, originating from the previously described phantom set, that ranged across all available optical properties.

The resolution of an SFDI system is typically determined for the absorption and reduced scattering coefficients separately through an edge response analysis, also known as a knife edge test, though the metric is underreported in the literature making it difficult to compare results.^20^^,^^45^^,^^46^ In this work, the edge response resolution was determined from contrast images obtained using the previously described set of epoxy resin phantoms. Using pairs of phantoms placed in contact with each other, which were matched in one property and varied in the other, a step response was generated. For the scattering contrast, both phantoms had very similar absorption coefficients at all four wavelength channels while having a 2× difference in their respective reduced scattering coefficients. Similarly, the absorption contrast was obtained using a pair of phantoms with the same reduced scattering coefficients and different absorption coefficients. Due to the differences in the absorption levels used for the phantoms, the absorption contrast varied among the different channels. The 850 and 625 nm channels had around a 2× difference among the absorption coefficients, whereas the 545 and 395 nm channels had around a 3× difference.

To minimize any air gaps between the two phantoms, which would introduce artifacts into the edge response, the phantom pairs were pressed together with a clamp during imaging. To fully assess the SFDI resolutions, contrast images were taken in a horizontal configuration, in which the edge response occurred in parallel with the direction of the patterned illumination, and a vertical configuration, in which the edge response was perpendicular to the illumination pattern. For each image, an 8.6  mm×25.8  mm region was assessed with the 25.8 mm spanning the two phantoms. To determine the edge response, the data within the region of interest were normalized to create a step function between 0 and 1. A generalized sigmoid function was then fit to the normalized data, and the result was used to determine the locations of the 10% and 90% contrast values. The resolution of the image was defined as the distance required for the edge response to go from 10% to 90% of the contrast, the typical metric used in edge response analysis.^47^ This process was repeated twice for the vertical configuration, with the edge at different locations within the field of view, and three times for the horizontal configuration, taking care to place the edge along different portions of the sinusoid patterns to account for any variation that may occur due to using a single-phase demodulation approach, with the results averaged together for each wavelength.

### 2.5 Quantitative Fluorescence

To validate the ability of the combined SFDI and fluorescence system to produce quantitative fluorescence images by correcting for the optical properties of the sample, a series of fluorescent phantoms were made. CPIII dihydrochloride (Santa Cruz Biotechnology, Dallas, Texas, United States) was used as the fluorophore at a fixed concentration of 5  μM to best match the excitation and emission properties expected during CPIII-mediated aPDT. As porphyrin absorption and fluorescence properties are known to vary significantly depending on the environmental conditions, epoxy resin phantoms were not used.^48^^,^^49^ Instead, liquid phantoms were made using dimethyl sulfoxide (DMSO) as the matrix component. The optical properties of these phantoms were varied using a slightly modified version of the broadband, multi-pigment phantom method.^41^ To aid in the homogenization of the added pigments into the DMSO, a less solid version of the acrylic paints was used (fluid acrylics, Golden Artist Colors, New Berlin, New York, United States). This limited the range of available pigments, and as such, only a single pigment was used to modulate each aspect of the optical properties. This included the scattering (PW4, zinc white), the absorption at the excitation wavelength of 395 nm (PY175, benzimidazolone yellow light), the absorption at the emission wavelength of 625 nm (PV15, ultramarine violet), and the baseline absorption (Super Black India Ink, Speedball). To provide for a wide range of raw fluorescence values, eight different DMSO phantoms were made using two different scattering levels and four different absorption levels, denoted in the name of each phantom using S and A, respectively. The absorption levels were further broken up into two different excitation absorption coefficients and two different emission absorption coefficients, as shown in Table 3.

**Table 3:** Target optical properties for the fluorescent liquid phantoms. Phantoms were made using two scattering levels (S) and four absorption levels (A).

	395 nm		625 nm	
	$\mu_{s}^{\prime }$ (${\mathrm{mm}}^{-1}$)	$\mu_{a}$ (${\mathrm{mm}}^{-1}$)	$\mu_{s}^{\prime }$ (${\mathrm{mm}}^{-1}$)	$\mu_{a}$ (${\mathrm{mm}}^{-1}$)
S1A1	6.00	0.60	3.75	0.05
S2A1	3.00	0.60	1.88	0.05
S1A2	6.00	0.60	3.75	0.10
S2A2	3.00	0.60	1.88	0.10
S1A3	6.00	1.80	3.75	0.05
S2A3	3.00	1.80	1.88	0.05
S1A4	6.00	1.80	3.75	0.10
S2A4	3.00	1.80	1.88	0.10

SFDI and fluorescence images were acquired for each phantom after pouring them into a non-fluorescent, epoxy resin container made to have tissue-like optical properties. SFDI images were acquired at 395 and 625 nm, following the previously outlined method, whereas the fluorescence images were acquired using the 395 nm treatment LED for excitation and imaged using the 620 nm imaging channel. Similar to the SFDI images, the exposure time for each fluorescence image was allowed to vary to make full use of the dynamic range of the detectors with the resulting images being normalized against their exposure times prior to analysis. In addition, the fluorescence images were corrected against the normalized reflectance of the 395 nm excitation light, taken on a 99% diffuse reflectance standard, to account for any spatial variations in the intensity. The resulting uncorrected fluorescence images for each of the phantoms were then converted into corrected fluorescence images using

$$f_{xm}=\frac{\mu_{a,x}}{(1-R_{x})R_{m}}F_{xm},$$ (2)

the method validated by Kim et al.,^50^ where $f_{xm}$ is the corrected fluorescence, $F_{xm}$. is the uncorrected fluorescence, $\mu_{a,x}$ is the absorption coefficient at the excitation wavelength, and $R_{x}$ and $R_{m}$ are the diffuse reflectance values at the excitation and emission wavelengths, respectively. To convert the resulting arbitrary units into a quantitative measure of fluorophore concentration, the corrected fluorescence images are compared with that of a reference with a known concentration. For the set of eight phantoms, each phantom was used as the reference inurn, with the concentration of CPIII being determined for the other seven within a 13  mm×13  mm region of interest. The resulting values were then compared with the expected 5-μM concentration by determining the MAPE. By averaging the errors that resulted from using each phantom as the reference, the overall potential error was estimated. This value was then compared with the one obtained from using the uncorrected fluorescence, in the same manner, to determine the improvement in accuracy afforded by the correction.

### 2.6 Bacterial Strains and Growth Conditions

The USA300 LAC strain of *S. aureus*, a community-acquired methicillin-resistant *S. aureus* strain, was used as the representative Gram-positive bacteria in this work. Bacteria were aerobically cultured on tryptic soy agar (TSA) at 37°C and 5% ${\mathrm{CO}}_{2}$ for 24 h. Overnight cultures were made by collecting and inoculating single colonies in 3 mL of tryptic soy broth (TSB) and growing under aerobic conditions in a shaking incubator (MaxQ 4450, Thermo Scientific, Waltham, Massachusetts, United States) at 294 rpm and 37°C for 18 h.

### 2.7 Photodynamic Therapy and Photobleaching Dosimetry

Stock solutions of 5-aminolevulinic acid hydrochloride (ALA, Sigma-Aldrich, St. Louis, Missouri, United States) were made in TSB immediately prior to use. After the incubation of the overnight cultures, subcultures were made by diluting 100  μL of USA300 into 900  μL of fresh media. To illicit different levels of photosensitization, the fresh media was made by varying the relative amount of the ALA stock solution and regular TSB to achieve final ALA concentrations of 0, 0.4, 1.0, and 4.0 mM. The subcultures were made in amber culture tubes to mitigate any potential unwanted light exposure and grown in a shaking incubator at 37°C overnight to ensure maximal conversion of ALA into CPIII. After incubation, the subcultures were centrifuged, and the pellets were washed with phosphate-buffered saline (PBS) before being recentrifuged and resuspended in PBS at the original subculture volume of 1 mL.

Using the bacteria at different levels of photosensitization, the ability of the imaging system to track the photosensitizer photobleaching during treatment was assessed. Resuspended subcultures were transferred individually to a single well of a 24-well plate. The well was then placed in the center of the field of view of the imaging system, with alignment being performed using the reflectance of the 850 nm channel to not risk an early initiation of PDT. Once positioned, the well was exposed to 25 min of the 395 nm treatment light, which at an average irradiance of $35\;\mathrm{mW}/{\mathrm{cm}}^{2}$, resulted in a total approximate light dose of $52.5\;J/{\mathrm{cm}}^{2}$. During the treatment, fluorescence images from the 620 nm channel were captured using a 200-ms exposure at a rate of one image per second. After the treatment, the fluorescence images were normalized to their exposure time, background subtracted, and spatially normalized to the intensity profile of the treatment light in the same manner as with the previously described phantom imaging. After corrections, a 200×200  pixel region of interest within the center of the well was selected, and the average value within the region of interest was determined for each captured frame.

To correlate the measured time-dependent fluorescence with the resulting efficacy of the PDT treatment, 30-μL samples were removed from the well during treatment at predetermined time periods and placed in the top row of a 96-well plate. Including a sample removed prior to light exposure to serve as a dark control, 12 samples were taken over the course of the treatment. In an attempt to provide a better estimate of the true dose response, shorter time intervals between samples were used in the early portions of the treatment, with the time from the start of the treatment being noted for each sample. Fluorescence frames that captured the plastic micropipette tip were removed from the final fluorescence time curves to prevent the plastic autofluorescence from impacting the fluorescence analysis. To determine the remaining concentration of viable bacteria, the samples were serially diluted 1:10 in PBS seven times, before plating 10  μL of each dilution on TSA. After growing in the dark at 37°C for 20 h, the final bacterial concentrations were estimated as the number of colony-forming units per milliliter (CFU/mL) by counting the number of colonies in the highest dilution with visible growth.

The time-dependent behavior of both the fluorescence signals and bacterial concentrations was assessed by fitting them to the single-exponential decay function

$$y(t)=a*e^{-t/\tau }+b,$$ (3)

where t is the amount of time since the start of the light exposure, and τ. is the associated time constant, indicative of the rate of change. The PDT results were fit over the first 10 min of the light dose, resulting in the time constants $\tau_{\mathrm{CFU}}$, whereas the fluorescence data were fit over the same period of time, excluding the first 15 s to omit the observed increase in fluorescence, resulting in the time constants $\tau_{F}$. To assess any correlations between the observed fluorescence behavior and the resulting PDT efficacy, Pearson correlation coefficients were determined among the concentration of ALA used, both of the time constants, the fluorescence intensity at the start of the treatment $F_{t=0}$, the maximum measured fluorescence $F_{\max}$, and the overall survival fraction (SF) of bacteria, which is defined as

$$\mathrm{SF}=\log_{10}(N/N_{0})$$ (4)

where $N_{0}$ is the bacterial concentration in CFU/mL of the dark control (t=0  s), and N. is the resulting bacterial concentration after the light exposure.

### 2.8 Longitudinal Fluorescence Spectroscopy

To begin to investigate the mechanism behind the dose-dependent fluorescence behavior observed at the start of the treatment, fluorescence spectroscopy was performed using *S. aureus* cultures photosensitized overnight with 4 mM of ALA using the same protocol described above. The washed cultures were placed underneath the 395 nm treatment light of the imaging system while inside clear centrifuge tubes. The resulting spectral emission was collected using a miniature spectrometer (Flame-S-VIS-NIR, Ocean Insight) placed adjacent to the sample. The spectra were collected continuously using a 100-ms exposure time for 60 s. Given an average irradiance of $28.7\;\mathrm{mW}/{\mathrm{cm}}^{2}$ at the sample, this correlated to an overall light dose of $1.72\;J/{\mathrm{cm}}^{2}$. As a comparison, the fluorescence spectra of a 1-μM concentration of CPIII dihydrochloride in DMSO were obtained following the same procedure.

Prior to analysis, the spectral response of the miniature spectrometer was calibrated using a National Institute of Standards and Technology (NIST) traceable white light source (UIS-LS, StellarNet, Tampa, Florida, United States), with each collected spectrum undergoing spectral response correction. After correction, the spectra were noise-filtered using a second-order Savitsky–Golay filter. To obtain just the porphyrin-specific fluorescence, the broadband autofluorescence arising from the bacteria and the centrifuge tube was approximated with a fourth-order polynomial fit, using an asymmetric truncated quadratic cost function, and subtracted from the original measurement.^51^ All spectra were truncated to the range of 580 to 720 nm for visualization and analysis. To compare the change in fluorescence intensity to that captured with the imaging system, each spectrum was summed and the change in fluorescence was calculated as

$$\Delta F/F=\frac{\left(F_{t}-F_{0}\right)}{F_{0}},$$ (5)

where $F_{t}$ is the fluorescence intensity at time t and $F_{0}$ is the fluorescence intensity of the first measurement.

## 3 Results and Discussion

### 3.1 Accuracy

**Fig. 3:**
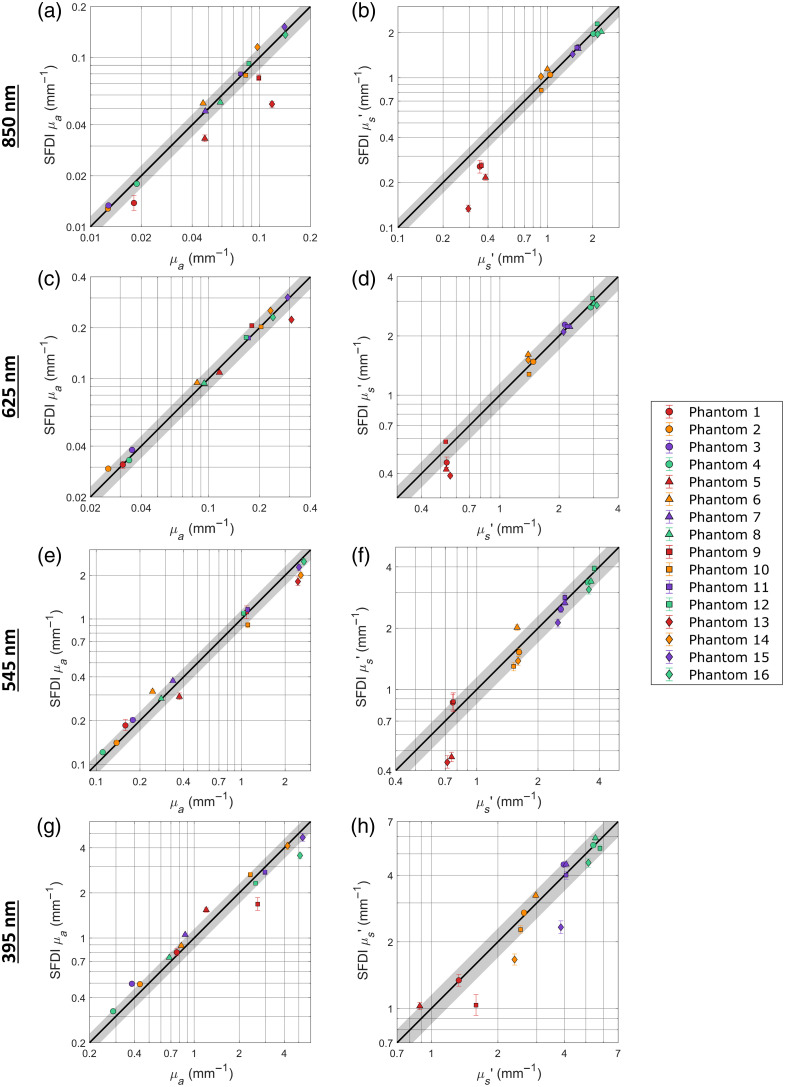
Comparative accuracies of the SFDI-derived optical properties. The SFDI-derived absorption (a), (c), (e), and (g) and reduced scattering coefficients (b), (d), (f), and (h) are compared with the ground truth properties at the 850 nm (a) and (b), 625 nm (c) and (d), 545 nm (e) and (f), and 395 nm (g) and (h) wavelength channels. Error bars represent the upper and lower quartiles of the measured properties within the analyzed region of interest. Shaded regions indicate a 15% error margin. Matching symbols represent phantoms made to have the same absorption coefficients, whereas matching colors represent phantoms made to have the same reduced scattering coefficients. Missing symbols indicate measurements that resulted in 100% undetermined pixels.

Figures 3(a)–3(h) compare the SFDI-derived optical properties of the 16 epoxy resin phantoms to their ground truth optical properties for the 850, 625, 545, and 395 nm channels, respectively. Overall, the SFDI system was found to have a 12.01% error for the absorption coefficient and a 12.15% error for the reduced scattering coefficients. This range of errors places the observed accuracy of the system within the expected range of other SFDI systems utilizing the SSOP methodology, despite the wider range of tested optical properties, with absorption coefficients extending over two orders of magnitude.^23^ Looking at the wavelength channels separately, the behavior of 850 and 625 nm show a high degree of similarity with the main difference being the larger errors at 850 nm for the four phantoms in the lowest scattering group. This is likely due to a slightly inaccurate model of the scattering behavior being utilized, and could be theoretically overcome using a reference phantom with a closer reduced scattering.^33^ If these four measurements are disregarded, the 850 nm MAPEs fall significantly, with the absorption coefficient error dropping from 12.96% to 6.55% and the reduced scattering coefficient error dropping from 13.73% to 5.85%. This brings the performance of the 850 nm channel closer in line to that of 625 nm, which had errors of 6.60% and 8.25%, respectively. The 395- and 545 nm channels, on the other hand, were found to be slightly less accurate overall, though still within the typical range for a single-phase SFDI system, despite utilizing the much greater than usual spatial frequency of $1.0\;{\mathrm{mm}}^{-1}$. Out of the two, the 545 nm channel performed slightly better overall, with an average absorption error of 12.88% and an average scattering error of 13.71%, as compared with the 15.58% and 12.90% observed for 395 nm. Unlike what was observed for 850 nm, no single group of phantoms led to these higher errors, indicating that the move to the higher spatial frequency was not without performance costs. Overall, the optical property maps supplied by the SFDI system should provide accurate determinations of tissue optical properties during PDT, including those of skin. This would allow for estimation of the variation in delivered light dose across the field of view during PDT and an accurate determination of the hemoglobin oxygen saturation.^32^^,^^52^^,^^53^

### 3.2 Precision

**Fig. 4:**
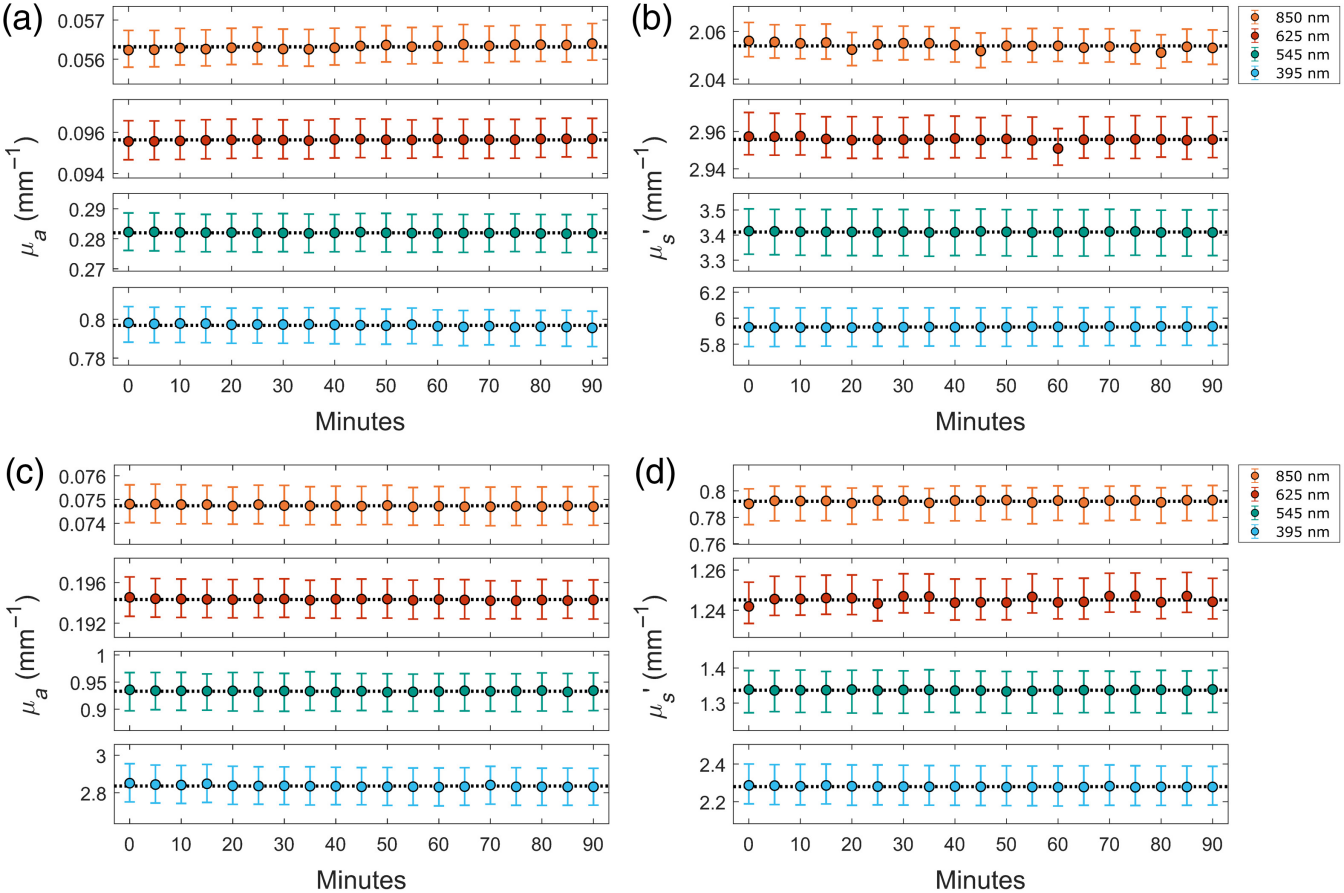
Drift test to measure the precision of the SFDI system. Representative results from a 90-min drift test show the stability of the measured absorption (a) and (c) and reduced scattering (b) and (d) coefficients at the four system wavelengths. Results shown are from phantom 8 (a) and (b) and phantom 10 (c) and (d). The dashed lines represent the means of the measurements over full-time course, whereas the error bars represent the upper and lower quartiles of the properties measured within the region of interest at each time point.

From the results of the drift tests, it was concluded that neither optical property, for any of the four wavelengths, experienced a continuous shift over time. Instead, all of the measurements were found to have some negligible and random variation around the time-averaged values. This can be observed in Fig. 4, which showcases the results of the drift test at all four wavelengths, for two representative phantoms. The average coefficients of variation for the measured absorption coefficients were found to be 0.11%, 0.05%, 0.14%, and 0.25% for the 850, 625, 545, and 395 nm channels, respectively. For the reduced scattering coefficients, these values were found to be similar at 0.10%, 0.08%, 0.14%, and 0.18%, respectively. These values are smaller than the spatial variation observed for each phantom by around an order of magnitude. Such small coefficients of variation indicate that, even for the samples with high absorption and low reflectance, the SFDI system produces stable optical property measurements over time, and thus, any significant changes that are observed can be accurately attributed to an actual change in the optical properties of the sample. With the stability being maintained over at least a period of 90 min, this conclusion should hold for nearly all variations of PDT treatment.

### 3.3 Resolution

**Fig. 5:**
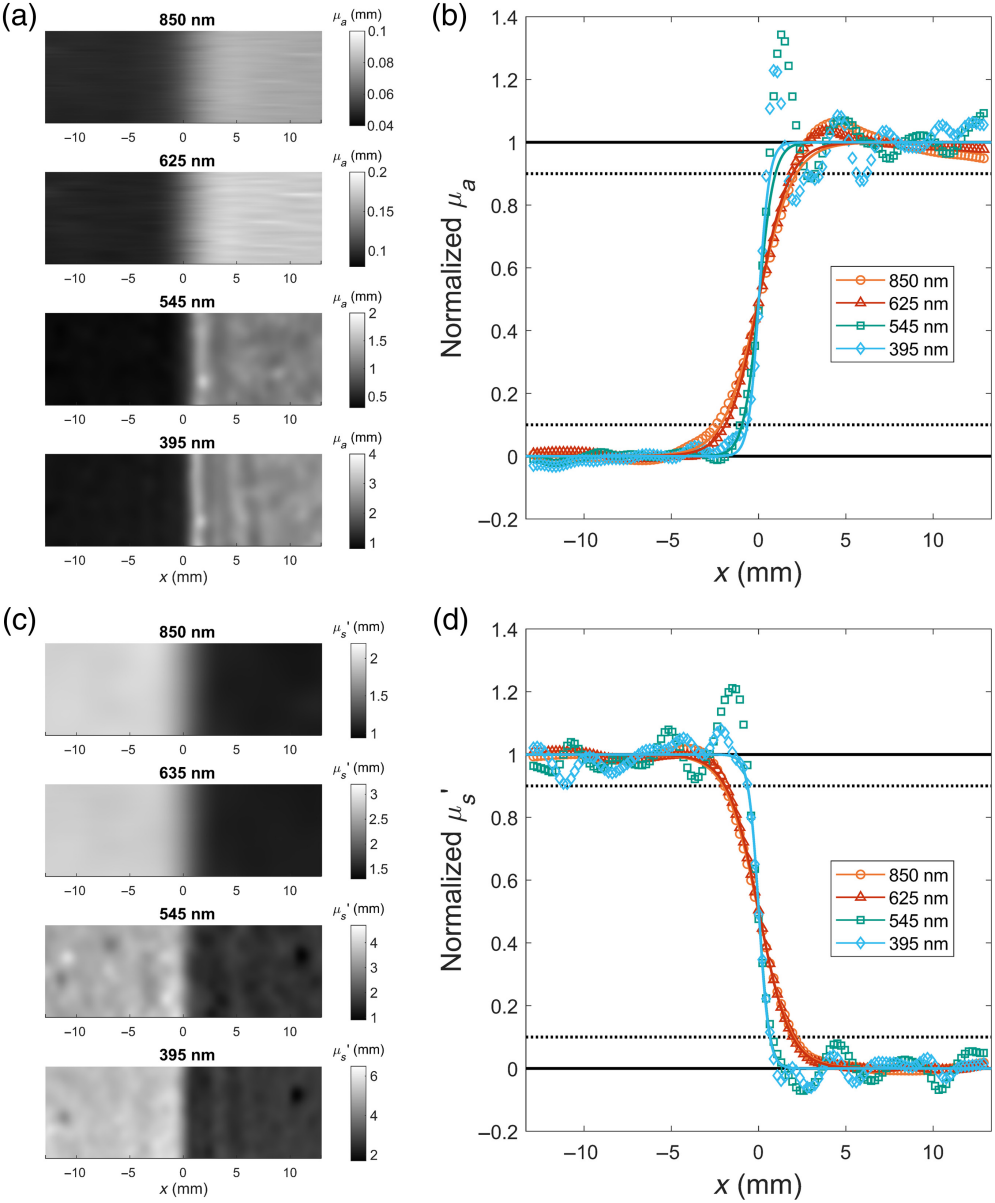
Edge response resolution of SFDI optical property maps. Horizontal edge responses of two adjacent phantoms with either a difference in the absorption coefficient (a) and (b) or in the scattering coefficient (c) and (d). (a) and (c) Representative images of the assessed region of interest. (b) and (d) Normalized edge response profiles for each wavelength. Symbols are the result of vertically averaging the region of interest and normalizing the results between 0 and 1, with every fifth pixel shown. Solid curves are the sigmoid functions fit to the full edge response image. Dotted black lines indicate the 10% and 90% contrast levels.

Figures 5(a) and 5(b) show representative horizontal edge responses of the SFDI system for the absorption coefficient, whereas Figs. 5(c) and 5(d) show the same for the reduced scattering coefficient. From the edge response profiles, the horizontal and vertical resolutions for each optical property and wavelength were determined, as summarized in Table 4. From the results, it can be seen that the resolution has a clear dependence on which spatial frequency is used, with the 395 and 545 nm channels, using a $1.0\text{-}{\mathrm{mm}}^{-1}$ spatial frequency, having a significantly better resolution than the 625- and 850 nm channels, which use a $0.3\text{-}{\mathrm{mm}}^{-1}$ pattern. As expected, the resolution was also found to be heavily dependent on the orientation of the boundary with respect to the illumination pattern, with the vertical orientation having a better resolution, on average.

**Table 4:** Horizontal and vertical resolutions for the single-phase SFDI optical property maps.

	Resolution (mm)			
	$\mu_{a}$		$\mu_{s}^{\prime }$	
	Horizontal	Vertical	Horizontal	Vertical
850 nm	4.74	2.15	3.75	2.55
625 nm	3.97	2.19	3.73	2.60
545 nm	2.14	1.72	1.51	1.09
395 nm	1.89	2.06	1.21	1.26

The most likely cause of both of these differences lies in the single-phase acquisition and the subsequent use of Fourier-domain filtering to achieve the demodulation of the captured images. Without stepping the projected pattern in phase, features on an unmoving sample will only experience a single portion of the sinusoidal period and are thus under-sampled. Any change in properties occurring in parallel with the spatial frequency direction (the horizontal orientation) will need to be physically large enough to produce a noticeable shift in the DC and AC envelopes independent of what portion of the illumination period it falls into. This issue is further compounded by the loss of spatial frequency information that arises from the filter-based demodulation. As both sets of filters primarily suppress spatial frequencies along the x-axis, the resolution along that direction is worse, resulting in the difference observed between the horizontal and vertical resolutions. The demodulation process for the $0.3\text{-}{\mathrm{mm}}^{-1}$ images utilizes an anisotropic bandpass filter to isolate the DC component, effectively serving as a low-pass filter. With the lower AC frequency and the relatively small field of view of the system, the required cutoff frequencies of this low-pass filter result in a significant loss of high-frequency information along the x-axis, resulting in the DC reflectance images having a worse resolution. With the filter preserving more of the high spatial frequency information along the orthogonal axis, the effect is not as strong for the vertical configuration, resulting in the larger discrepancy in resolution between the two orientations observed for 850 and 625 nm.

Although the spectral filtering used for the $1.0\text{-}{\mathrm{mm}}^{-1}$ spatial frequency images had a less significant effect on the loss of spatial frequency information, it is not without its own negative effects. As can be seen from Figs. 5(b) and 5(d), both the 395 and 545 nm edge responses exhibit an overshoot of the 100% contrast level, ranging from a 10% to a 40% overshoot, depending on where the edge fell with respect to the spatial illumination period. Considering the edge responses for the corresponding DC and AC reflectance images, it was determined that these overshoots occur because of a difference in their respective resolutions. With a sharper relative resolution in the DC reflectance, the measured reflectance pairs change at different rates. This results in instances where, on either end of the boundary, the DC reflectance has already reached its new value while the AC reflectance is still changing. The resulting mismatched reflectance pairs give rise to incorrect optical properties that overshoot the actual values. Although it was not observed for the pairs of contrast phantoms tested here, this effect could even result in a number of undetermined pixels due to the DC reflectance temporarily dropping below the AC reflectance. Although the overshoots can be suppressed by applying an additional low-pass filter to the DC reflectance images, this would come at the cost of decreasing the overall resolution.

Overall, the spatial resolutions for the different channels fall into an acceptable range as the lack of fine features is not expected to overly impact the determination of PDT dosimetry, which would mainly care about large, bulk changes within the field of view. However, as mentioned previously, it is difficult to truly compare the performance of the system due to the lack of similarly characterized SFDI systems. It appears that no other SFDI systems using the SSOP approach have reported resolution values, so the relative impact of the different image filters used in this work cannot be determined. When compared with the multi-phase SFDI systems that have reported SFDI resolutions, the system used in this work is found to be on the lower end of the scale.^20^^,^^45^^,^^46^ However, the reported values across these systems are relatively inconsistent, with reduced scattering resolutions ranging from 50  μm up to 2 mm. Although the use of a multi-phase demodulation approach would be expected to improve the spatial resolution of the images by an unknown degree, it would come at a known cost to the improved temporal resolution provided by imaging only a single phase.

### 3.4 CPIII Quantitative Fluorescence

**Fig. 6:**
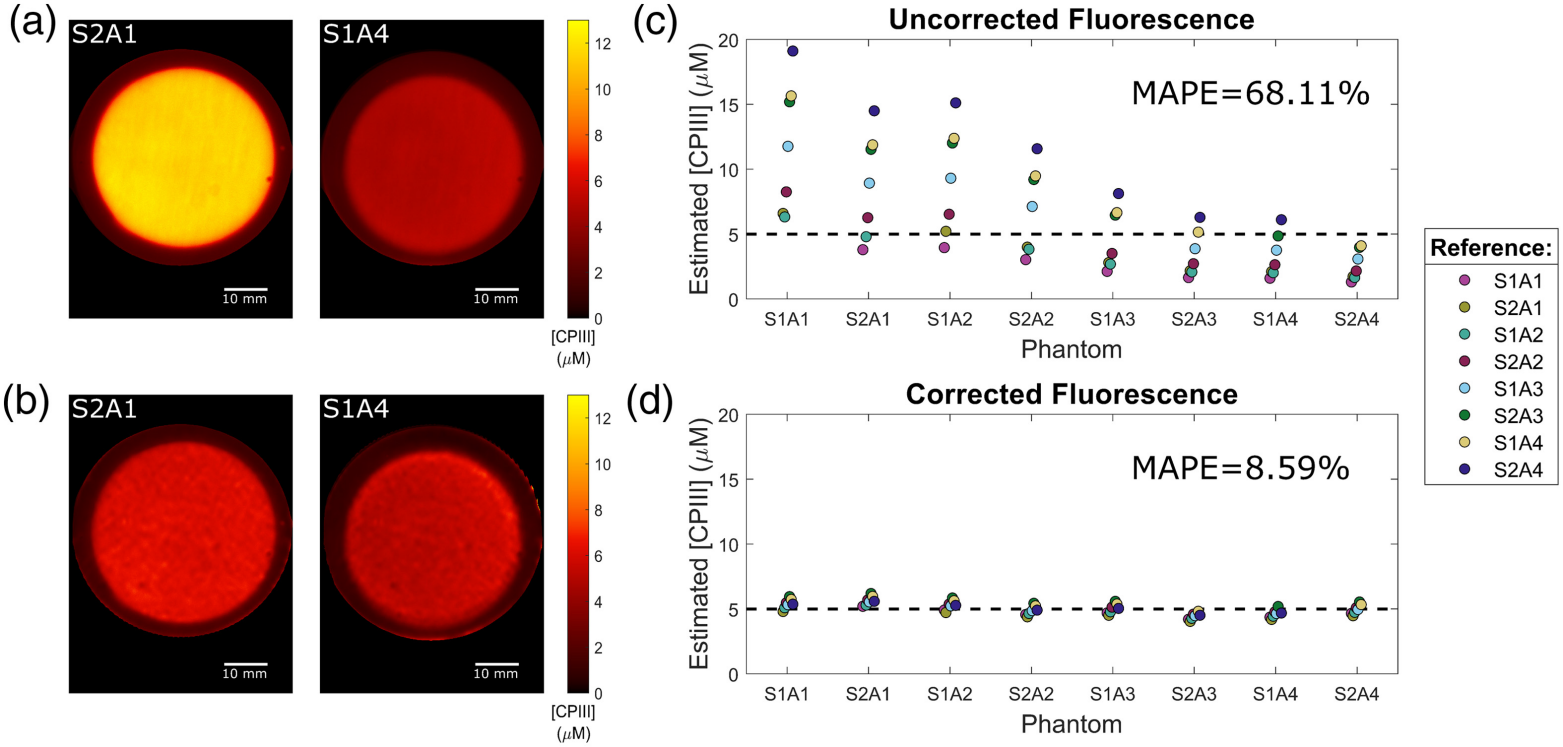
Quantitative fluorescence of CPIII-containing liquid phantoms. Liquid phantoms with equal fluorophore concentrations (5  μM) demonstrate a significant difference in their uncorrected fluorescence (a) and (c), due to the differences in their relative absorption and scattering. Correcting the fluorescence (b) and (d), using the optical properties at the excitation and emission wavelengths, equalizes the relative fluorescence intensity across the phantoms. (a) and (b) Representative images of predicted CPIII concentration using the uncorrected and corrected fluorescence of phantoms S2A1 and S1A4, using phantom S2A3 as the reference. (c) and (d) Estimated concentration of CPIII for each liquid phantom when using each of the other seven phantoms as the known reference. Using uncorrected fluorescence results in significant deviations from the true concentration (dotted line) as compared with the corrected fluorescence, as demonstrated by an MAPE of 68.11% and 8.59%, respectively.

With the general performance of the SFDI portion of the imaging system characterized, the ability to leverage the optical property maps to perform more complex, multi-wavelength measurements needed to be assessed, with the most prevalent of such measurements being that of quantitative fluorescence. Figures 6(a) and 6(c) show the estimated concentration of CPIII for the eight different liquid phantoms using the uncorrected fluorescence images. The collected fluorescence from the eight liquid phantoms was found to be highly variable, despite including the same concentration of CPIII, due to the varying optical properties at both the 395 nm excitation wavelength and the 620 nm emission wavelength. An increase in the scattering of the media resulted in an increase in the emitted fluorescence, whereas an increase in the absorption, at either wavelength, resulted in the opposite.

Using any of the phantoms as a reference to estimate the fluorophore concentration in the other seven results in highly inaccurate results, with the largest absolute error observed being an overestimation of the 5  μM CPIII concentration by over 13  μM. By analyzing the estimation error across all the reference combinations, an overall MAPE of 68.11% was obtained for the set. Although this in itself is a significant level of error, it is likely an underestimate of the overall potential error, as all of the reference samples still had relatively similar optical properties to the samples under test. If a reference was used that did not include the high absorption at the excitation wavelength, the resulting fluorophore concentrations would likely be significantly underestimated.

In contrast to the uncorrected results, Figs. 6(b) and 6(d) showcase the estimated CPIII concentrations for the same eight phantoms using the fluorescence images that have been corrected using Eq. (2). By accounting for the combination of excitation and emission optical properties, the variability among the measured corrected fluorescence values was greatly reduced, resulting in a MAPE for the predicted fluorophore concentration of only 8.59% across all of the reference combinations. This represents an ∼8 times improvement in the accuracy of estimating the concentration of CPIII, as compared with directly using the uncorrected fluorescence. This level of error is comparable to similar values reported in the literature, despite the phantoms used in this work having a much greater absorption at the excitation wavelength.^26^ This indicates that the slightly higher error observed in the accuracy of the optical properties for the 395 nm channel did not overly impact the correction factor or the resulting quantitative fluorescence of CPIII. Hence, we could conclude that the system can be expected to accurately determine physiologically relevant concentrations of porphyrin fluorescence in a variety of different tissue environments.

In addition, the resolution of the excitation absorption coefficient map was found to be sufficient to not noticeably affect the clarity of the boundary between the CPIII fluorescence of the liquid phantoms and the autofluorescence of the surrounding epoxy resin container. It can be observed, however, that the demodulation noise present in the 395 nm absorption coefficient map does impact the corrected fluorescence image quality by imparting a slight pattern to the image, increasing the spatial variance. Across all eight phantoms, it was found that the correction approximately doubled the standard deviation across the region of interest, with the corrected images reaching an average standard deviation of 0.23  μM. However, as this is still less than the 0.35-μM standard deviation across the means of the different phantoms, it is not expected to overly impact the accuracy of any fluorophore concentration measurements.

### 3.5 aPDT Photobleaching Dosimetry

**Fig. 7:**
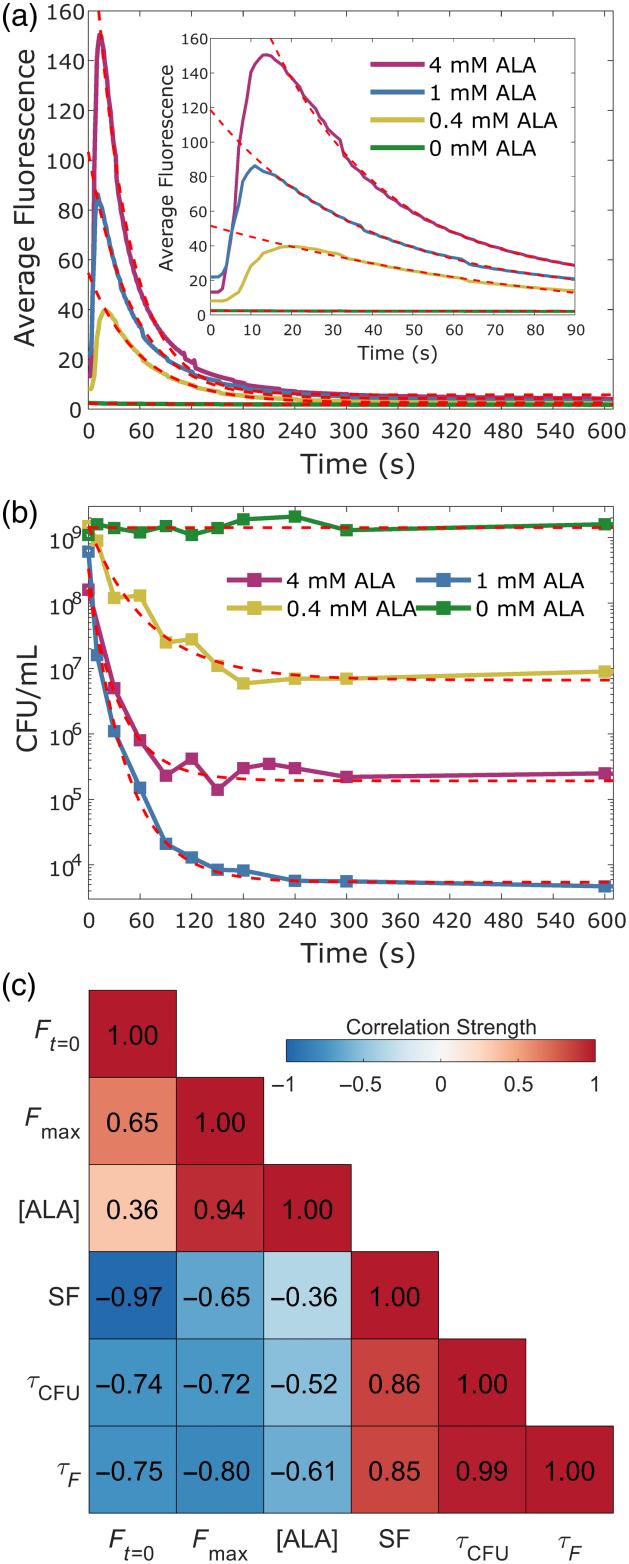
Preliminary photobleaching dosimetry of endogenously photosensitized *S. aureus*. (a) After an initial increase in fluorescence over the first 10 to 15 s of the light treatment, a characteristic photobleaching response was observed for all photosensitized samples, which was fit to a single exponential decay function. A similar decay curve was fit to the (b) measured time-dependent efficacy of the PDT treatments. (c) Treatment and fluorescence responses were compared through a series of Pearson correlation coefficients.

After fully characterizing the combined system, its ability to measure and monitor the fluorescence induced during aPDT was determined. Figure 7(a) shows the captured fluorescence dynamics for *S. aureus* photosensitized using 4, 1, and 0.4 mM of ALA, as well as a sample with 0 mM to serve as a control. As expected, the control sample showcased minimal fluorescence with all three photosensitized samples having a greater amount of measured fluorescence at the start of the light exposure, though with values not directly tracking the amount of ALA used. Interestingly, after the initialization of the light exposure, the measured fluorescence of the photosensitized samples increased by a significant degree, instead of the expected photobleaching behavior. Over the first 10 to 15 s of the treatment [see Fig. 7(a) subset], the fluorescence showed between a 4- and 11.4-times increase in intensity, after which the expected photobleaching decay was observed. Despite the atypical fluorescence behavior, the PDT dose response appeared to be consistent throughout the light treatment, as seen in Fig. 7(b), with a strong effect being observed over the initial 60 s. This indicates that the photochemistry resulting in the rapid fluorescence increase is occurring in parallel with the photodynamic effect.

Using the time-dependent responses, an assessment of the correlation between the photosensitizer fluorescence and PDT efficacy was performed. As can be seen in Fig. 7(c), the three fluorescence parameters and two PDT parameters selected were all found to have relatively strong correlations with each other, with two in particular standing out. The strongest correlation was found between the time constants for the fluorescence, $\tau_{F}$, and PDT, $\tau_{\mathrm{CFU}}$, exponential decay fits, with a Pearson correlation coefficient of 0.99. Looking at the time constants themselves, it was found that the decay rates of the two curves within an ALA concentration were on the same scale, with $\tau_{F}$. values of 59.9, 50.6, and 39.1 s and $\tau_{\mathrm{CFU}}$ values of 70.5, 43.6, and 38.8 s for the 0.4-, 1-, and 4-mM concentrations of ALA, respectively.

The next highest correlation was found to be between the overall survival fraction of the bacteria and the initial fluorescence at the start of the treatment, with a correlation coefficient of −0.97. What is interesting is that the peak fluorescence, which better correlated with ALA concentration, had a much lower correlation with PDT efficacy at −0.65. What should be noted, however, is that the concentration of viable bacteria, expressed as CFU/mL, before the start of the light treatment was lower for the 4-mM ALA sample [Fig. 7(b)], having almost 1-log less bacteria as compared with the samples cultured with 1 mM or less of ALA. This may indicate that either the amount of ALA or, more likely, the amount of CPIII produced after the long subculture was toxic to the bacteria, even in the absence of light, resulting in a change in the response to the light treatment. Alternatively, this may be an indication that the cause of the increase in fluorescence competes with the PDT process as 4 mM of ALA resulted in a max ΔF/F of 10.4, whereas 1 and 0.4 mM only had max increases of 2.9 and 3.9, respectively.

**Fig. 8:**
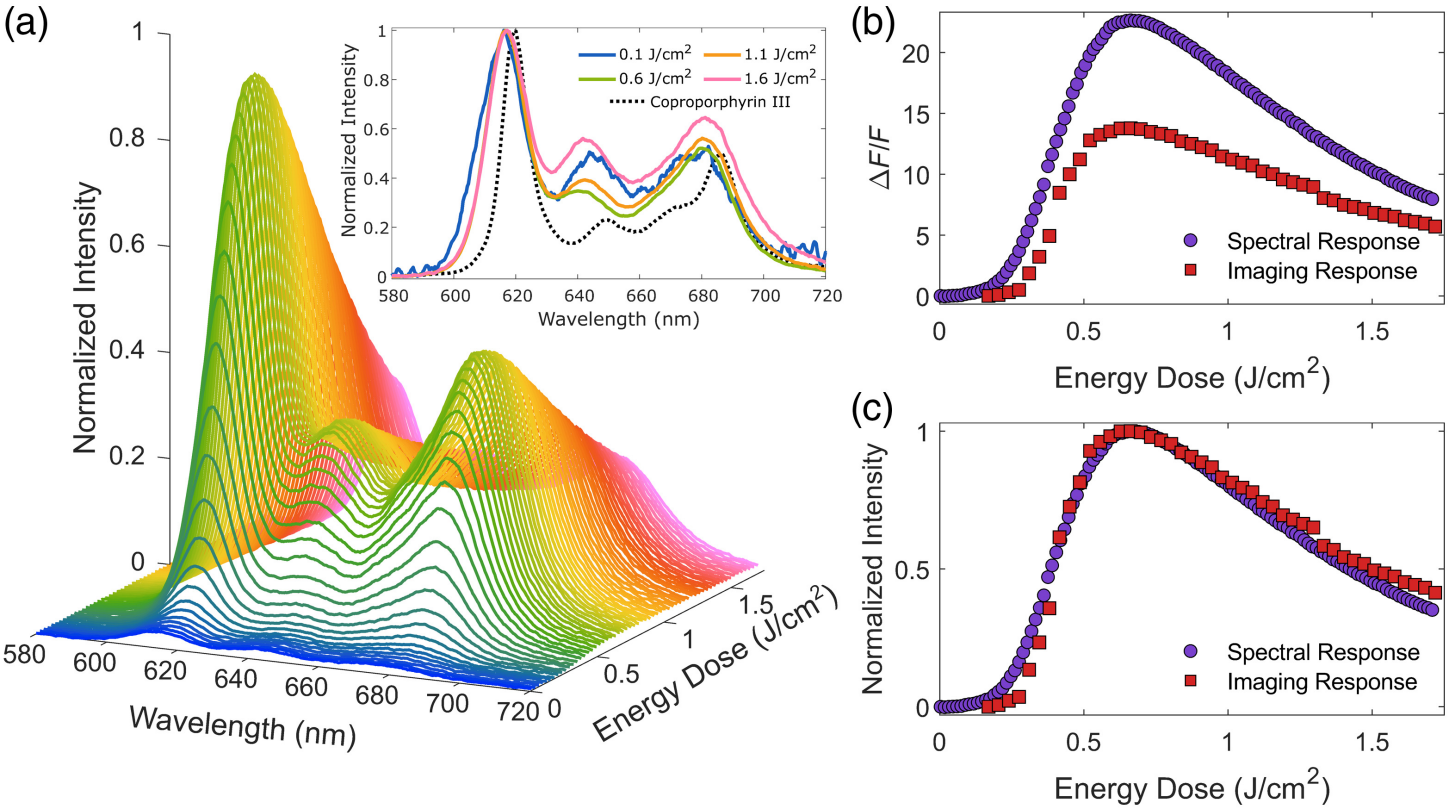
Longitudinal fluorescence spectroscopy of *S. aureus* photosensitized overnight with 4-mM ALA. (a) Relative spectral intensity of the porphyrin fluorescence throughout $1.7\;J/{\mathrm{cm}}^{2}$ of 395 nm light normalized to the overall max intensity. Although the overall intensity experiences large fluctuations, the spectral shape remains mostly stable, except for the relative intensity of the 640 nm peak (a) (inset) which decreases during the fluorescence increase and increases during the photobleaching. The change in fluorescence intensity (b) occurs in a dose-dependent manner, with the porphyrin-specific signal experiencing a majority of the change in fluorescence. Normalization of the fluorescence change (c) showcases that the dose-dependent response is the same for both the imaging and spectral acquisitions.

It is currently unknown why the initial increase in fluorescence is observed, primarily due to the current lack of understanding of CPIII photochemical properties. Informed by the behavior of protoporphyrin IX (PPIX), we hypothesize that one potential mechanism is that the increase in fluorescence is due to the formation of a CPIII-derived photoproduct that has an increased fluorescence yield as compared with CPIII itself. The formation of photoproducts is a well-understood process for PPIX, with each product having distinct emission profiles which, under the right filters, can lead to an increase in the measured fluorescence.^12^^,^^54^ However, there have been no reports of such a significant increase in fluorescence during PDT using PPIX. Looking at the longitudinal emission of the photosensitized *S. aureus* [Fig. 8(a)] shows that the fluorescence maintains its spectral shape throughout the fluorescence increase and into the photobleaching period. The only major spectral shift observed is with the 640 nm emission peak, commonly associated with porphyrin photoproduct. The relative intensity of this peak decreases during the fluorescence increase, further disabusing the photoproduct hypothesis, before increasing throughout photobleaching, as expected. Finally, with the red channel of the imaging system having a similar dose-dependent fluorescence response as that of the full spectrum [Fig. 8(c)], despite the magnitude of the fluorescence increase being lower due to the presence of autofluorescence in the images [Fig. 8(b)], we can conclude that the fluorescence increase itself is not due to the spectral sampling of the imaging bandpass filter. However, there do appear to be some differences in the rising and falling edges of the two responses which are likely due to photoproduct dynamics, as visualized by the changes in the 640 nm emission peak.

With the emission spectra remaining CPIII-like throughout the light exposure, there are two other potential mechanisms for the increase in fluorescence that can be hypothesized. The first is that the light treatment is inducing a change in the chemical environment of the CPIII which is altering the fluorescence properties. It has been shown for PPIX that the local environment heavily influences both the absorption and emission profiles of porphyrins.^48^^,^^49^ In this regard, the PDT could be causing the bacteria to lyse, freeing sequestered CPIII and introducing it to an environment that enables either increases in the fluorescence yield or a shift in the excitation better aligned with 395 nm light. The other potential mechanism hypothesizes that the majority of ALA is not fully converted into CPIII and is instead stored as its precursor coproporphyrinogen III. If this is true, light exposure would result in photooxidation events, producing new fluorescent CPIII molecules from the minimally fluorescent precursor and providing an explanation for why the fluorescence spectra maintain the same shape throughout the change in intensity. Overall, although more data will be necessary to truly characterize the photobleaching dosimetry behavior of CPIII-mediated aPDT, these preliminary results showcase the capability of the developed imaging system to perform real-time PDT dosimetry.

## 4 Conclusion

In this work, a combined fluorescence and SFDI imaging system was developed using inexpensive, off-the-shelf components, with the capability of monitoring the explicit and implicit dosimetry of endogenous CPIII-mediated aPDT during non-contact treatment. The combined system was shown to be capable of performing single-frame SFDI at four wavelengths across the visible and near-infrared spectrum, with the resulting optical property maps having both high accuracy and precision over time. Although the Fourier-filtering approach to the demodulation of the patterned illumination resulted in a reduction in the resolution of the optical property maps, the performance was maintained within that of systems using traditional demodulation approaches while achieving video rate acquisition speeds. For all phantoms with reflectance values similar to that of the skin, the total acquisition time for the set of SFDI images fell below 1 s, despite high absorptions at 395 and 545 nm increasing the variable exposure times used. With SFDI processing times ranging from 100 ms down to 0.5 ms per frame, depending on the implementation, SFDI dosimetry parameters can be obtained in real time.^24^ The optical property maps produced by the system were shown to be capable of accurately correcting fluorescence images against the optical properties of the surrounding medium, resulting in an 8× improvement in determining the fluorophore concentration as compared with using the uncorrected fluorescence. Finally, the system was shown to be sensitive enough to accurately track the photobleaching dynamics of photosensitized *S. aureus* during PDT, with initial comparisons to the treatment outcome identifying a number of highly correlated features including the dose–response time constants. With the ability to accurately monitor both spatially and temporally resolved PDT dosimetry, without additional equipment or interrupting the treatment, this system lightens the burden of detailed treatment monitoring, increasing the potential of a more thorough clinical adoption of individualized PDT dosimetry.

## Data Availability

All relevant code, data, and materials are available from the authors upon request from the corresponding author.
